# Leiomyosarcoma of the Renal Vein Mimicking a Primitive Renal Cell Carcinoma: Case Report of an Unusual Presentation

**DOI:** 10.1155/2021/6637533

**Published:** 2021-05-12

**Authors:** Amal Fekkar, Hafsa Elouazzani, Ahmed Jahid, Kaoutar Znati, Fouad Zouaidia, Zakia Bernoussi

**Affiliations:** Department of Pathology, Ibn Sina University Hospital Center, Rabat, Morocco

## Abstract

Primary leiomyosarcomas (LMS) of vascular origin are rare tumors, and more than half of the cases arise in the inferior vena cava (IVC). Primary LMS of the renal vein are extremely rare tumors with only a few cases reported in the literature. Their diagnosis is made only by pathological features. Histologically, they are made of atypical spindle-shaped cells arranged in long intersecting fascicles. Tumor cells stain positive for myogenic markers in immunohistochemistry. Standard treatment consists of radical nephrectomy followed by chemotherapy and/or radiotherapy. Because of insufficient histological data and follow-up, the prognosis factors are not well identified. Overall prognosis of renal vein LMS is poor. We report here an exceptional case of a huge LMS of the right renal vein mimicking a primitive renal cell carcinoma, occurring in a 56-year-old male patient.

## 1. Introduction

LMS are rare tumors accounting for about 11% of all newly diagnosed soft tissue sarcomas [1]. LMS arising from a vascular channel represent a rare group of tumors with only a few hundred cases reported in the literature [2]. They more often arise from the inferior vena cava, far less commonly from the pulmonary artery, and rarely in systemic arteries [2]. Primary LMS of the renal vein are rare with only 67 cases reported worldwide [3]. They occur predominantly in females with a peak in the fifth and sixth decades [4, 5].

Clinical diagnosis of renal vein LMS is difficult because of nonspecific symptoms [4]. The radiological features are nonpathognomonic and do not allow adequate differential diagnosis with other retroperitoneal solid tumors or renal cell carcinoma (RCC) [6]. The diagnosis is made by pathological features. We present an exceptional case of a huge LMS of the renal vein occurring in a 56-year-old male patient. The singularity of this case lies in the huge size of the tumor and its unusual presentation showing a preponderant intrarenal component rather orienting towards a primitive RCC extending into the renal vein, underlining the importance of a careful anatomopathological examination and exposing some diagnostic challenges.

## 2. Case Report

A 56-year-old male, a chronic smoker (11 pack/year), presented with 5-month history of right lumbar region pain, intermittent hematuria, and weight loss. His past medical history was unremarkable. Clinical examination revealed a bimanually palpable right flank mass.

**Figure 1:**
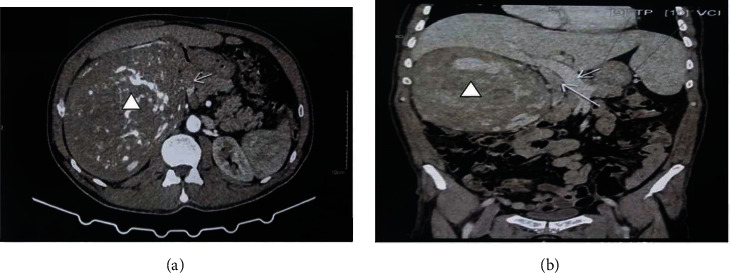
Abdominal computerized tomography scan showing (a) a heterogeneous mass occupying the right kidney (arrowhead). (b) The mass seems to arise from the renal parenchyma (arrowhead) and to extend into the renal vein lumen while compressing the inferior vena cava (arrow).

An abdominal computerized tomography (CT) scan showed a 25 × 11 × 8 cm heterogeneous mass occupying the right kidney and extending into the renal vein lumen while compressing the IVC (Figure 1). The rest of the abdominal cavity was unremarkable. A CT scan of the thorax showed nodular lesions that may correspond to a secondary location of his tumor.

The other laboratory findings were normal.

Thus, a right radical nephrectomy with the resection of the thrombus of the right renal vein was performed.

**Figure 2:**
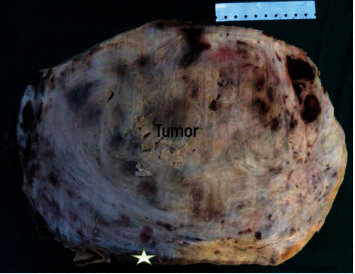
Gross examination of the tumor showed a gray-white whorled appearance with foci of hemorrhagic and necrotic changes. Presence of a peripheral rim of the normal kidney (star).

In the laboratory, the gross examination revealed a huge encapsulated mass weighing 2300 g and measuring 27 × 13 × 9 cm. The cut surface was gray-white showing a whorled appearance with foci of hemorrhagic and necrotic changes. The tumor was attached to the renal vein wall. A portion of the tumor occupied the renal vein lumen, but most of this huge tumor extended beyond the hilum area compressing the normal renal parenchyma (Figure 2).

**Figure 3:**
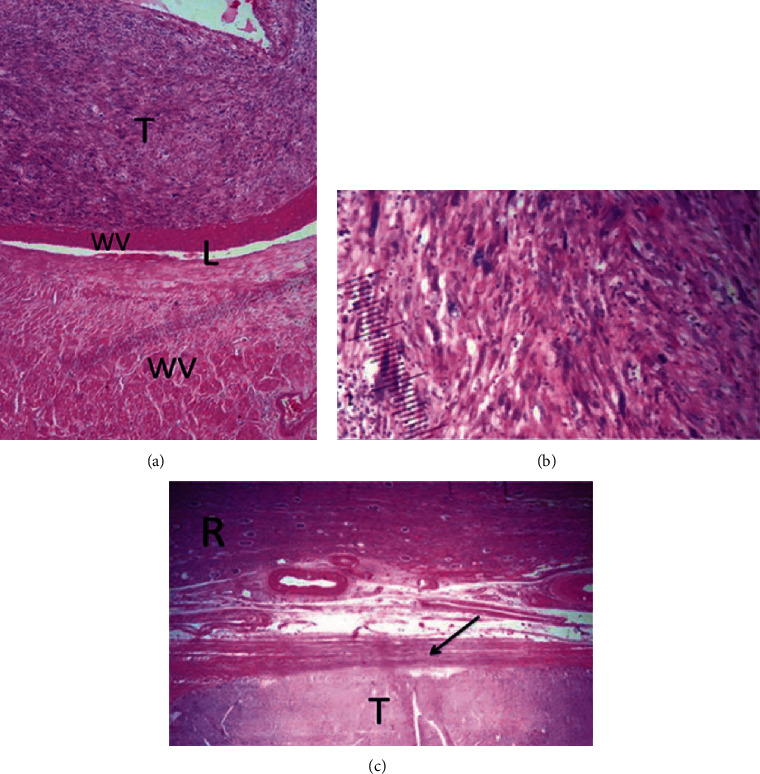
Hematoxylin and eosin staining. (a) A spindle cell neoplasm arranged in alternating fascicles arising from the wall of the renal vein. (b) The cells are spindle shaped with elongated blunt-ended nuclei, coarse chromatin, and eosinophilic cytoplasm. Presence of pleomorphic cells showing marked nuclear pleomorphism, hyperchromasia, and prominent nucleoli. (c) The tumor is encapsulated (arrow) compressing the residual renal parenchyma. T: tumor; L: vascular lumen; WV: wall of the renal vein; R: renal tissue; arrow: tumor capsule.

Microscopic examination showed an encapsulated tumor composed of interlacing fascicles of spindle-shaped cells with elongated blunt-ended nuclei, coarse chromatin, and moderately abundant eosinophilic cytoplasm, intermingled with pleomorphic cells presenting marked nuclear pleomorphism, hyperchromasia, and prominent nucleoli. The mitotic count was 8 per 10 high-power fields with 3 atypical mitoses. Areas of tumor necrosis were present. A portion of the wall of the renal vein was sarcomatous in continuity with the tumor. There was a capsule separating the tumor from the normal renal parenchyma. The renal vein's surgical resection margin was free of tumor (Figure 3).

**Figure 4:**
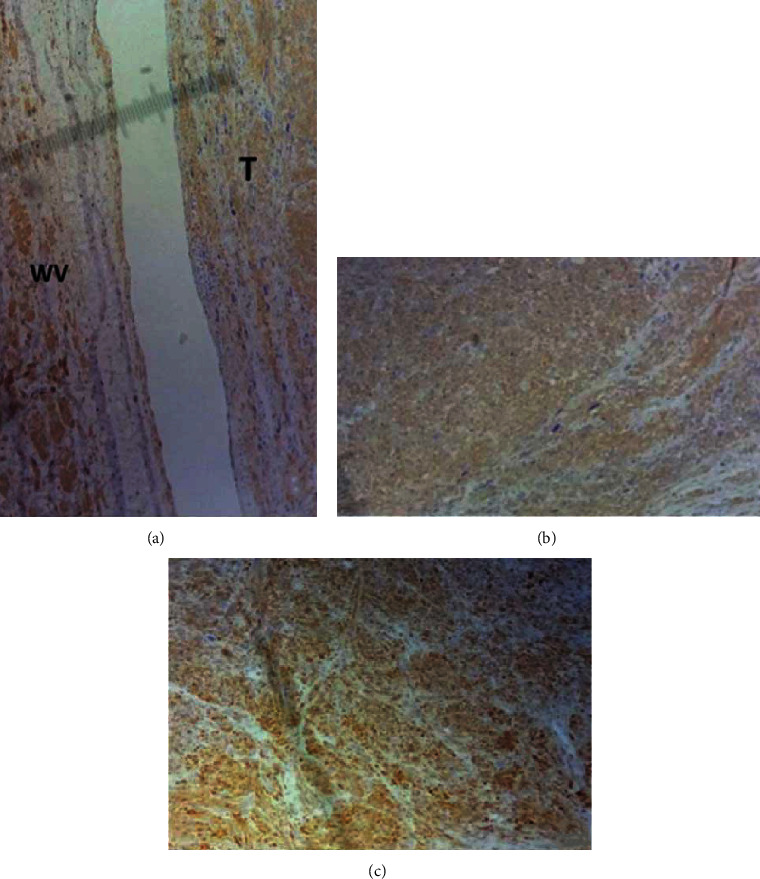
Immunohistochemical study showing diffuse positivity for smooth muscle actin (a), H-caldesmon (b), and desmin (c).

In the immunohistochemical study (Figure 4), the tumor cells showed diffuse positivity for smooth muscle actin (SMA), heavy chain caldesmon (H-caldesmon), and desmin. They were negative for cytokeratin AE1/AE3, epithelial membrane antigen (EMA), CD34, melan A, and HMB45.

The pathological diagnosis of leiomyosarcoma of the renal vein, grade II of the FNCLCC (the National Federation of Centers for the Fight Against Cancer) and pT4NxMx (UICC, 8th edition 2017), was therefore retained.

After surgery, the patient's general condition was very impaired and no neoadjuvant treatment was administered. The patient passed away one month later.

## 3. Discussion

LMS of vascular origin are an uncommon group of tumors [2]. The most common site is IVC (70% of all vascular LMS) [7]. Primary renal vein LMS are exceptional. Their clinical and imaging features can significantly overlap with those of advanced primary renal neoplasms, particularly renal cell carcinoma with venous extension [8]. In the 4^th^ edition of the *WHO Classification of Tumours of the Urinary System and Male Genital Organs*, LMS of the renal vein is considered an entity of renal LMS [9].

A few cases of LMS of the renal vein have been reported so far [5, 7, 10]. It is more frequent in women (3F/1M), the mean age of presentation is 57 years (range 27–88 years), and it is predominantly located in the left side (60%) [10]. Various theories have been suggested regarding this clinical presentation. Female preponderance is supported by the theory that estrogenic stimulation leads to growth and proliferation of smooth muscle tumors [5]. The more frequent involvement of the left renal vein is suggested by its longer length compared to the right renal vein [5, 7]. In our case, the patient was a male, and the tumor involved rather the right renal vein.

Clinically, vascular LMS have diverse symptoms determined by the location of the tumor, rate of growth, and degree of collateral blood flow or drainage in the affected part [2]. Renal vein LMS usually have an insidious presentation, with signs and symptoms occurring at late stages of the disease [11]. Clinical symptoms are nonspecific and do not differ from another renal tumor. The most common are abdominal pain, weight loss, and palpable abdominal mass [4, 10].

Imaging studies of LMS (magnetic resonance imaging (MRI) and contrast-enhanced CT) are nonspecific but helpful in delineating the relationship to adjacent structures, particularly in the retroperitoneum [1]. The nonpathognomonic radiological features of these tumors do not allow adequate differential diagnosis with respect to other retroperitoneal solid tumors or renal cell carcinoma with invasion of the renal vein [6]. CT scan appearance is generally that of a homogeneous, well-circumscribed, solid mass with minimal contrast enhancement in the region of the renal hilum. MRI demonstrates usually a well-defined lesion in the renal hilum characterized by an isointense signal compared with the kidney on T1-weighted images and slightly increased signal intensity on T2-weighted images, albeit less intense in comparison with the kidney [4]. In our case, given its huge size, the tumor extended outside the region of the hilum and replaced a big portion of the renal parenchyma that was compressed in the periphery what made the diagnosis more challenging.

The diagnosis is made by the pathological features. Grossly, LMS are large, solid, gray-white, soft to firm, and variably necrotic [9]. They usually generate the displacement of structures rather than invasion [1]. Microscopically, the morphological features of renal vein LMS are identical to those of LMS arising at other sites. Typical LMS shows spindle-shaped cells with plump, blunt-ended nuclei and moderate to abundant, pale to brightly eosinophilic fibrillary cytoplasm. The cells are set in long intersecting fascicles parallel and perpendicular to the plane of section. Moderate nuclear pleomorphism is usually noted, although pleomorphism may be focal, mild, or occasionally absent. Mitotic figures, including atypical ones, are typically easy to find. Tumor cell necrosis is often present in larger tumors [1]. Low-grade tumors resemble differentiated smooth muscle cells but with increased cellularity, cytological atypia, and mitotic activity. High-grade tumors are pleomorphic, requiring immunohistochemical stains and adequate sampling to distinguish from other malignancies such as sarcomatoid carcinoma with leiomyosarcomatous differentiation and other pleomorphic sarcomas [9]. Immunohistochemically, at least one myogenic marker (SMA, desmin, or h-caldesmon) is positive in 100% of cases, and >70% of cases are positive for more than one of these markers. None of these is absolutely specific for smooth muscle, and positivity for two myogenic markers is more supportive [1]. The majority of LMS are negative for cytokeratin, epithelial membrane antigen, CD34, and S100. HMB45, melan A, myogenin, and MyoD1 are negative [9].

Leiomyosarcoma of the renal vein must be differentiated from sarcomatoid renal cell carcinoma extending into the renal vein as both of them exhibit similar clinical, radiological, and pathological features, but their prognosis and treatment modality are different [11]. The sarcomatoid RCC is not a distinct histological subtype; it is defined by the sarcomatous transformation of the RCC, characterized histologically by a transformative growth pattern of the epithelial neoplasm into malignant spindle-shaped cells in variable proportions, with marked nuclear atypia and prominent mitotic figures. The extensive sampling of the tumor in search of epithelial elements and the immunohistochemical stains are very helpful to distinguish this entity from LMS of the renal vein [11]. In the sarcomatoid RCC, the epithelial markers (CKAE1/AE3, EMA) stain positive and myogenic markers (such as smooth muscle actin, H-caldesmon, and desmin) are negative, while in the LMS, there is no epithelial component and the myogenic markers are positive. In our case, despite adequate sampling, the tumor was negative for epithelial markers (CKAE1/AE3, EMA) and diffusely positive for myogenic markers. Immunohistochemical stains are also very helpful to distinguish pleomorphic LMS from other pleomorphic sarcomas.

In some cases, a low-grade variant of LMS requires to be distinguished from a leiomyoma. True leiomyomas arising from vessels are rare, and this diagnosis should be made with extreme caution and only after the lesion has been sampled extensively [2]. In our case, the diagnosis of malignancy was obvious considering the presence of tumor necrosis, cellular pleomorphism, and mitoses.

Due to their varying appearance and pleomorphism, renal epithelioid angiomyolipomas can mimic other benign or malignant tumors. In our case, the immunohistochemistry was valuable in ruling out this diagnosis as the tumor cells were negative for melanocytic markers (HMB45, melan A).

In our case, the diagnosis of LMS was made on the basis of morphological and immunohistochemical results, but the huge size of the tumor (27 cm in the greatest dimension) made it difficult to elucidate its origin and to understand its behavior, especially since the radiological features were not very helpful. According to the 4th edition of the *WHO Classification of Tumours of the Urinary System and Male Genital Organs*, the LMS of the kidney can arise from the renal capsule, the renal parenchyma, the pelvic muscular wall, or the main renal vein. Careful macroscopic examination and extensive sampling allowed us to confirm that the tumor arose from the wall of the renal vein and to determine its mode of extension. The gross examination showed that the tumor was in continuity with the wall of the renal vein. The microscopic examination confirmed this finding and showed that a portion of this wall was sarcomatous (Figure 3). Giving that LMS tends to replace and compress without true infiltration, it is unlikely that the tumor had arisen from the renal parenchyma or the renal capsule and then infiltrated the wall of the renal vein and filled the lumen. In addition, there was a capsule separating the tumor from the normal renal parenchyma compressed in the periphery in accordance with the usual mode of extension of LMS.

The gold standard treatment consists of radical nephrectomy followed by chemotherapy and/or radiotherapy. Complete surgical removal remains the only curative therapy [9]. Unfortunately, because of the lack of large systematic case series, no role of postoperative chemotherapy or radiotherapy has been determined [12].

Overall prognosis of renal vein LMS is poor [9]. Local recurrence is reported in 40% of the cases, and distant metastases are primarily to the lungs, followed by the liver and bones [10, 11]. However, it has been reported that tumors originating from IVC show a more aggressive course than those arising from the renal vein [5, 13]. Studies performed at Memorial Sloan Kettering, New York, showed that the major prognostic factor is total surgical resection. When it is complete, 5-year survival free of disease is of approximately 60%, vs. just 30 to 35% when it is partial. Once total removal is performed, the major prognostic factor becomes histological grade, with 5-year disease-free survival of 90 to 95% for low-grade tumors and of 30 to 35% for high-grade tumors [14]. In some series, the size of the tumor is >3 cm and the mitotic rate are predictive of local recurrence and metastasis [10].

A recent study of prognostic factors shows that in univariate analysis, factors predictive of overall survival are surgical margins, while factors predictive of local recurrence free survival are IVC luminal extension and grade. No factors predictive of distant metastasis free survival were identified [3]. It remains difficult to evaluate the true overall survival rate, as most reports do not have long follow-ups.

## 4. Conclusion

LMS of the renal vein is a rare and aggressive tumor. Its ominous nature requires timely diagnosis and treatment. The diagnosis is made only by pathologic features. However, sometimes it is extremely challenging to differentiate LMS of the renal vein from sarcomatoid RCC with extension into the renal vein lumen and to determine the origin of the tumor. The prognosis of advanced renal LMS is poor, and the appropriate treatment is still controversial given the extreme rarity of this neoplasm.

## Abbreviations

LMS: Leiomyosarcoma
IVC: Inferior vena cava
RCC: Renal cell carcinoma
CT: Computerized tomography
SMA: Smooth muscle actin
H-Caldesmon: Heavy chain caldesmon
FNCLCC: The National Federation of Centers for the Fight Against Cancer
MRI: Magnetic resonance imaging.
